# Primary Cutaneous Gamma-Delta T Cell Lymphomas: A Case Series and Overview of the Literature

**DOI:** 10.3390/dermatopathology8040054

**Published:** 2021-11-17

**Authors:** Silvia Alberti-Violetti, Carlo Alberto Maronese, Luigia Venegoni, Valentina Merlo, Emilio Berti

**Affiliations:** 1 UOC Dermatologia, Fondazione IRCCS Ca’ Granda, Ospedale Maggiore Policlinico, 20122 Milan, Italy; carlo.maronese@unimi.it (C.A.M.); valentina.merlo@policlinico.mi.it (V.M.); emilio.berti@unimi.it (E.B.); 2 Department of Pathophysiology and Transplantation, University of Milan, 20122 Milan, Italy; luigia.venegoni@unimi.it

**Keywords:** gamma-delta, cutaneous lymphoma, subcutaneous, epidermotropic

## Abstract

Primary cutaneous gamma-delta T cell lymphomas (PCGDTCLs) are rare and aggressive cutaneous malignancies that have been diagnostically challenging for dermopathologists and clinicians since their first published descriptions in 1991. Since then, the availability of immunostaining for T cell receptors γ and δ in formalin-fixed paraffin-embedded samples has greatly increased our knowledge of the gamma-delta phenotype by showing that it may also be present in the context of indolent entities, such as mycosis fungoides (MFs) and lymphomatoid papulosis, and this has raised questions concerning its diagnostic and prognostic implications. We here describe the histological and clinical differences between the dermo-epidermal and subcutaneous sub-groups of PCGDTCL observed in a cohort of 20 patients attending a single experienced centre, with particular focus on cases with an MF-like presentation, which are still less well defined than those of classic MF.

## 1. Introduction

According to the WHO classification of cutaneous neoplasms [1], primary cutaneous gamma-delta T cell lymphomas (PCGDTCLs) are rare and aggressive entities characterised by the clonal cutaneous proliferation of activated mature gamma-delta T cells with a cytotoxic phenotype. The first cases of PCGDTCL were published in 1991 by Berti et al. [2], who described a case of disseminated pagetoid reticulosis characterised by an epidermotropic gamma-delta infiltration, and Burg et al. [3], who described a case characterised by T cell receptor (TCR) δ-positive subcutaneous plaques without any signs of epidermotropism. PCGDTCLs usually affect the cutaneous extremities of adults of either sex, frequently spreading to mucosa and extra-nodal sites but generally sparing lymph nodes, spleen, and bone marrow [1].

The existing definition does not capture the full spectrum of gamma-delta lymphoproliferative cutaneous disorders, as gamma-delta subtypes of mycosis fungoides (MFs) and lymphomatoid papulosis, although recognized within the WHO classification, lay outside of its boundaries. The increasing availability of immunomarkers of the δ chain of the TCR in formalin-fixed paraffin-embedded (FFPE) tissue samples over the last few years has extended our knowledge of gamma-delta cutaneous proliferations by showing that such infiltrates can also be present in typical alpha/beta lymphoproliferative disorders, in which they play a reactive rather than a malignant role [4]. However, this finding has also raised questions concerning their prognostic value as their clinical course may also be more indolent than the classic course of PCGDTCL [2,5,6].

The updated 2017 WHO classification divided PCGDTCLs into predominantly epidermal, dermal, and subcutaneous forms on the basis of the location of the neoplastic infiltrate, and highlighted differences in their clinical courses [1]. However, cases of MF with a gamma-delta phenotype are still poorly characterised and have usually only been considered in the context of MF, although some authors disagree [1,5,7].

Diagnosing PCGDTCLs remains a challenge for dermatologists, pathologists, and hematologists and has important consequences for patient management. We here describe a cohort of PCGDTCL patients attending a single tertiary referral centre to show that there are histological and clinical differences within the same diagnosis, and also evaluate the clinical and prognostic implications of various pathological markers, including the expression of Vδ1 and Vδ2.

## 2. Materials and Methods

### 2.1. Patient Selection

After obtaining Institutional Review Board approval, we retrospectively reviewed the records of PCGDTCL patients treated and followed up at the Dermatological Clinic of the University of Milan (Italy), all of whom satisfied the clinical and/or histo-pathological criteria defined in the WHO classification [1]. The data considered were age at the time of diagnosis, sex, clinical features, treatment, follow-up, the date of the last follow-up examination, and outcome, including the cause of any deaths.

### 2.2. Histology and Immunohistochemistry

FFPE sections of skin biopsies were histologically examined in order to evaluate the pattern and composition of the infiltrate. 

The large panel of immunohistochemistry tests included CD3, CD4 (Dako, Glostrup, Denmark), CD5 (Novocastra, Newcastle upon Tyne, UK), CD7, CD8, CD30, CD45RO, CD45RA (Dako), CD56 (Novocastra), CCR4 (Abcam, Cambridge, UK), TIA-1 (Beckman Coulter, Indianapolis, USA), granzyme B, perforin (Novocastra), BetaF1 (Thermo Fisher, Waltham, USA), TCR Vδ1, TCR Vδ2 (Beckman Coulter), TCR*γ* (Thermo Fisher), and MIB-1 (Dako).

### 2.3. TCR Gene Rearrangement

Polymerase chain reaction analyses of the *TCRγ* gene were made using the BIOMED2 protocol [8] and DNA extracted from formalin-fixed tissues using the QIAamp DNA mini kit (Qiagen, Hilden, Germany).

### 2.4. Statistical Analysis

The Kaplan–Meier method was used to determine the median overall survival (OS), which was defined as death due to any cause. Cox’s proportional hazards regression analysis and 95% confidence intervals (CIs) were used to assess potential prognostic factors.

## 3. Results

The study involved 20 Caucasian patients with PCGDTCL and a median age at diagnosis of 62 years (range 46–77). The male/female ratio was 1:2. Table 1 summarises their clinical data.

The patients were divided into two groups on the basis of the pathological presentation of the neoplastic infiltrate: dermo-epidermal or subcutaneous.

**Figure 1:**
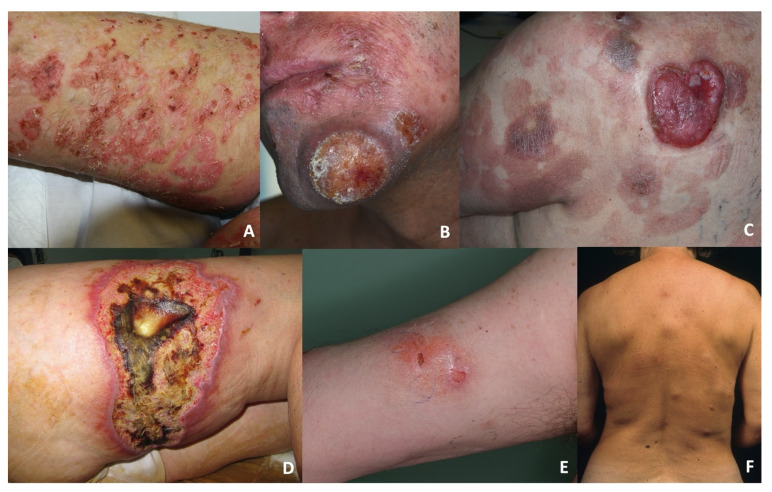
(**A**) A DE-GDTCL patient presenting with pagetoid reticulosis characterised by diffuse superficial patches and plaques. (**B**) An ulcerated tumour on the face of a DE-PCGDTCL patient. (**C**) A tumour associated with MF-like plaques on the back of a DE-PCGDTCL patient. (**D**) Pyoderma gangrenosum-like plaques on the leg of a S-PCGDTCL patient. (**E**) A deep erythematous plaque on the arm of an S-PCGDTCL patient. (**F**) Multiple deep nodules on the back of an S-PCGDTCL patient.

The dermo-epidermal group (DE-GDTCL) consisted of nine patients (45%): two with diffuse pagetoid reticulosis (RP, previously designated as Ketron–Goodman disease) characterised by diffuse superficial erythematous plaques with erosions and crusts (Figure 1A), and seven with ulcerated nodules and tumours, which were associated with diffuse ulcerated plaques in four cases (Figure 1B). Four patients reported a previous history of early stage mycosis fungoides (MFs) lasting a median of 36 months (range 28–48): one of the classic CD4+ variant, two of the folliculotropic CD4+ variant, and one of the CD8+ variant with folliculotropism (Figure 1C). Two of these patients died of PCGDTCL progression after 5 and 36 months; the other two are still alive after 12 and 84 months of follow-up (the individual tumours of the former were successfully treated with local radiotherapy, but she still has indolent folliculotropic MF plaques). 

The subcutaneous group (S-GDTCL) consisted of 11 patients (55%): two with an unusual clinical presentation characterised by ulcerated plaques resembling pyoderma gangrenosum (Figure 1D), and nine presenting with diffuse violaceous deep nodules and plaques mainly on the lower limbs and trunk that progressed to ulceration and necrosis (Figure 1E,F). One patient reported refractory celiac disease. 

Follow-up data were available for 17/20 patients. At the time of the last follow-up, 13 patients (65%) had died of the disease or complications during treatment, and one of the patients with the RP variant died of causes unrelated to the disease only three months after diagnosis. Three patients (15%) are alive with the disease: two DE-GDTCL patients, and one with subcutaneous plaques on the legs and abdomen whose indolent course is surprisingly different from that of typical PCGDTCL. This patient has been treated with systemic steroids and low-dose cyclophosphamide, and now has stable disease without any signs of extra-cutaneous involvement after 60 months of follow-up.

The median OS of the patients as a whole is 12 months (95% CI 5–228): 36 months (95% CI 3–228) for those with DE-GDTCL, and 12 months (95% CI 3–60) for those with S-GDTCL, with no statistically significant difference between the two subgroups (*p* = 0.37).

Most of the patients (12/20, 60%) underwent single- or multi-agent chemotherapy; phototherapy and immunomodifying treatments, such as interferon alpha or bexarotene, were reserved for the patients with early phase DE-GDTCL. Individual tumours were frequently treated with local radiotherapy. Only one patient received brentuximab vedotin. One DE-GDTCL patient with multiple plaques and nodules underwent allogeneic stem cell transplantation (alloSCT) but experienced a cutaneous relapse during the following year, and died 11 years after transplantation due to the infective complications of a lung transplantation for pulmonary graft versus host disease.

## 4. Pathological Data and Immunophenotyping

**Figure 2:**
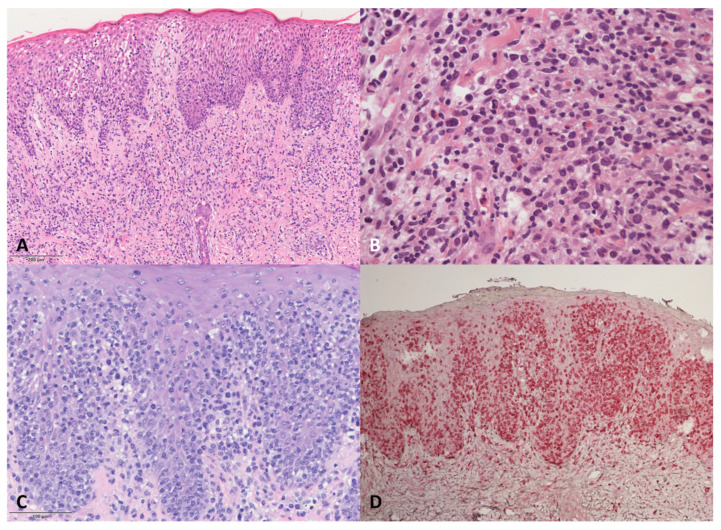
Histological features of the DE-GDTCLs. (**A**) Medium-to-large pleomorphic cells with hyper-convoluted nuclei infiltrating the epidermis and the superficial dermis (plaque). Haematoxylin and eosin (H&E), 100×. (**B**) Nodular infiltrate with medium-to-large pleomorphic neoplastic cells showing mitotic figures mixed with eosinophils and blasts. H&E, 400×. (**C**) Marked epidermotropic infiltrate of medium-sized hyperchromatic lymphocytes and some blasts. H&E, 200×. (**D**) TCRδ staining of the lesions shown in C. Immunophosphatase, 200×.

DE-GDTCL skin samples (Figure 2) showed dermal infiltrates associated with epidermotropism in all but two patients, who were both characterised by a dense neoplastic infiltrate in the dermis, with one tending to show subcutaneous involvement. Necrotic keratinocytes were frequent, but no Pautrier micro-abscesses were observed. One of the two patients with RP was characterised by a pagetoid neoplastic infiltrate mainly localised to the epidermis; the other had gamma/delta CD45RA+ cells mainly in the epidermis, associated with a lichenoid pattern of reactive CD45RO+ cells in the upper dermis.

The pathological features of the four DE-GDTCL patients with a previous history of MF were particular. During the MF phase, one had a superficial lichenoid CD4+ infiltrate with epidermotropism, whereas the other three showed a superficial infiltrate of small/medium-sized cells with marked folliculo- and epidermotropism (two with a CD4+ and one with a CD8+ immunophenotype). On the contrary, the PCGDTCL tumours were characterised by the almost complete loss of epidermo- and folliculotropism. Cytotoxic markers were negative in all cases, but some blasts were observed. The neoplastic cells were double negative for CD4 and CD8 and expressed TIA1 in all four cases. MF phase samples were available for further study in two cases, and showed that the CD4+ and CD8+ alpha/beta neoplastic cells were mixed with a small population of gamma/delta CD45RA+ cells in the epidermis during the phase of MF *en plaque*. In both cases, we found the same TCRδ clone in both the MF and PCGDTCL samples.

**Figure 3:**
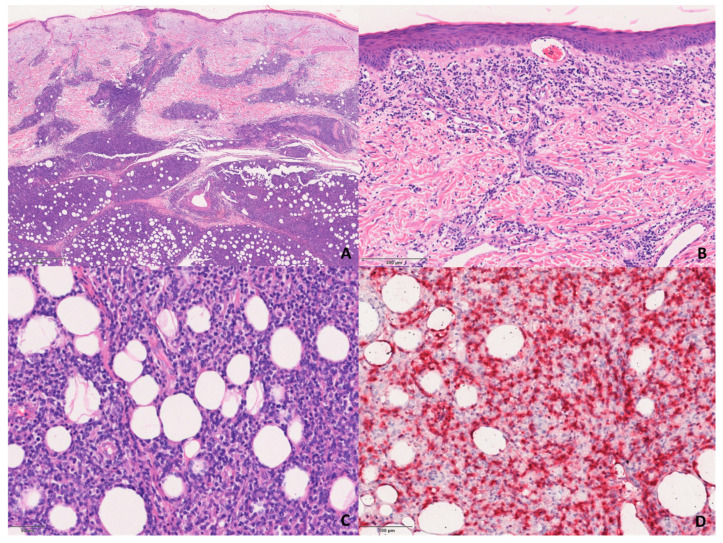
Histological features of the S-GDTCLs. (**A**) Dermal and subcutaneous involvement of the infiltrate. H&E, 200×. (**B**) Detail of the infiltrate in the dermis. H&E, 200×. (**C**) Detail of the lobular infiltrate of pleomorphic lymphocytes in subcutaneous tissue. H&E, 200×. (**D**) TCRδ-positive neoplastic cells in subcutaneous tissue. Immunophosphatase, 200×.

The S-PCGDTCL cases showed the diffuse proliferation of pleomorphic lymphocytes with prominent involvement of subcutaneous tissue and dermis (Figure 3). The infiltrate tended to focally rim adipocytes in some cases, and all but two showed intra-epidermal vesiculation, necrosis, and ulceration.

Immunophenotypically, all of the cases were double-negative for CD4 and CD8, except for three that were CD8+.

Four of the 17 tested patients were positive for CD45RA: three in the DE-GDTCL and one in the S-GDTCL group. As previously mentioned, it is interesting to note that the two DE-GDTCL patients who had a small population of CD45RA+TCRδ+ in the epidermis of their initial MF lesions lost CD45RA expression during PCGDTCL progression.

TCRδ expression was found in 17 of the 20 patients; two of the remaining three cases were positive for TCRγ but not for TCRδ, and one had a TCR-silent phenotype that had changed during tumour progression from a previous MF-like phase characterised by CD8+ alpha/beta cells mixed with a small population of CD45RA+TCRδ+ cells in the epidermis.

TCR-Vδ1 and Vδ2 expression was evaluated in 11 patients (four DE-GDTCL and seven S-GDTCL). Vδ1 was positive in four cases (three DE-GDTCL and one S-GDTCL), and Vδ2 was expressed in seven (six S-GDTCL and one DE-GDTCL).

CD5 was tested in 16 patients, and proved to be negative in 10 (62.5%), five in each subgroup. There was no significant correlation with survival (*p* = 0.12)

Low-medium CD30 expression (5–15%) was found in seven (41%) of the 17 patients tested, and also did not significantly correlate with survival (*p* = 0.13).

Eight of the 15 patients tested expressed CD56, which again did not significantly correlate with survival (*p* = 0.16) even though it was mainly positive in S-GDTCL patients (including the case characterised by an indolent course); it was also found in one DE-GDTCL patient with dense dermal infiltrate without epidermo-tropism.

CCR4 was negative in six of the seven DE-GDTCL patients tested: the seventh showed CCR4 positivity both on the MF-like plaques and the non-epidermotropic tumour during PCGDTCL progression (patient #19).

## 5. Discussion

Over the last few years, the introduction of TCRδ testing of FFPE samples has led to an increase in the number of case reports of cutaneous gamma-delta proliferations, thus showing that a gamma-delta phenotype may also be present in many lymphoproli-ferative disorders other than PCGDTCL and may lead to different prognostic outcomes [5,6,9]. Observations concerning numerous cases of PCGDTCL have shown that the disease can have a clinico-pathological presentation and clinical course that are different from those contained in the classic description, and raised questions concerning the existence of “indolent” PCGDTCLs. However, the use of such potentially misleading terminology is only based on findings of small reactive gamma/delta-positive T cell populations in infiltrates and/or short periods of follow-up [4].

Our study evaluated patients with a diagnosis of PCGDTCL in order to assess their clinical and histological characteristics and whether these correlate with the expression of TCR Vδ1 and TCR Vδ2. The patients were divided into groups on the basis of the prevalently dermo-epidermal or subcutaneous localisation of the neoplastic infiltrate, which has long been demonstrated to underlie the prognostic heterogeneity of PCGDTCLs. Merrill et al. [10] retrospectively described a series of 13 patients with epidermotropic GDTCLs (EGDTCLs, defined as those having >75% of lymphoma cells within the epidermis) and seven with dermal/subcutaneous involvement, and found that mortality was lower in the former group (3/13 vs. 5/7). This indicated clinical and histopathological similarities with MF and, in fact, the final diagnosis of these patients was gamma-delta MF. Interestingly, the EGDTCLs tended to be in a more advanced stage and have a worse prognosis than typical MF, and the patients tended to have smaller cells, less cellularity, and a reduced proliferation index than their dermal/subcutaneous counterparts [10]. We also found a difference in the survival of our DE-GDTCL and S-GDTCL patients, although it was not statistically significant probably because of the small sample size. 

Our clinical results partially confirm the findings of Guitart et al. relating to a large series of 53 PCGDTCL patients attending various American centres [11]. These authors described three subsets of clinico-pathological presentation: (i) classic panniculitis-like deep indurated plaques on the extremities; (ii) single or multiple lesions resembling pyoderma, cellulitis, hematoma, or an arthropod bite reaction at one anatomical site; and (iii) an MF-like presentation characterised by a less aggressive course and the presence of non-activated gamma-delta cells with minimal or absent TIA-1 and granzyme B expression. Some of these cases progressed to a more aggressive phase consisting of ulcerated plaques, tumours, and the typical expression of cytotoxic markers, with one case changing from a CD4+ immunophenotype to a CD8+ phenotype.

In a subsequent paper, Daniels et al. [7] described 42 PCGDTCL patients, 16 of whom had been previously diagnosed as having MF on the basis of their clinical and histo-logical features. A subset of these developed ulcerated treatment-resistant tumours that were clinically, genetically, and transcriptionally indistinguishable from PCGDTCL. On the basis of the recent WHO classification [1], these patients were diagnosed as having gamma-delta MF with PCGDTCL-like progression, but, given that this progression in gamma-delta MF cases is not predictable, the authors suggested classifying all cutaneous gamma-delta lymphomas together [7]. In our patients with an initial diagnosis of MF that progressed to PCGDTCL with nodulo-tumoral lesions, we compared the histological and immunophenotypic features of the first indolent phase with those of the subsequent aggressive progression and, although adequate specimens were available for only two cases, documented a complete immunophenotypic shift with the loss of CD4 or CD8 expression, the expansion of the TCRδ+ population or TCR silencing, and the expression of TIA1 and granzyme B. 

Such findings are also not uncommon during the progression of this and other lymphomas [5]. Agbay et al. [12] identified a change of at least one antigen in nine cases of progressive PCGDTCL and, as in our patients, identically sized monoclonal rearrangements of TCRγ were maintained during progression. The authors concluded that the immunophenotypic changes did not seem to be related to changes in the T cell clone, and suggested that antigen modulation may be involved in the pathogenesis of PCGDTCLs. The finding of a TCR silent phenotype in one of our patients is an uncommon feature that was probably due to TCR instability, which has not only been reported during the progression of cutaneous gamma-delta lymphomas, but also during the progression of hepatosplenic gamma-delta T cell lymphomas, enteropathy-associated T cell lymphomas evolving from refractory celiac disease, and other alpha-beta lymphomas [13].

On the basis of the landmark findings of Daniels et al. [7], which demonstrated that PCGDTLs arise from two different originating cells depending on the tissue compartment involved even if they have similar genomic landscapes, we assessed TCRVδ1 and Vδ2 expression in FPPE cutaneous biopsy samples. According to Daniels et al., lymphomas arising from the outer cutaneous layer originate from Vδ1 cells (the predominant gamma-delta cells in the epidermis and dermis), whereas panniculitic lymphomas arise from Vδ2 cells, the predominant gamma-delta T cells in fat [7,14]. Unsurprisingly, our findings were in line with those of Daniels et al. [7], and showed the predominant expression of TCR Vδ1 in DE-GDTCLs and TCR Vδ2 in S-GDTCLs. Interestingly, the DE-GDTCLs expressing TCR Vδ1 also included an MF-like case that progressed to PCGDTCL with tumours, and the only case of DE-GDTCL expressing Vδ2 was characterised by a dense dermal infiltrate without epidermotropism and short survival, which is biological behaviour more similar to that of S-GDTCLs.

As expected, CD5 expression was frequently lost in our patients and did not significantly correlate with either subgroup or survival. A large review of 246 PCGDTCLs published by Kamijo et al. [15] claims that CD5 positivity and the absence of cytotoxic markers are positive prognostic factors, but we did not find any correlation with survival.

The published data concerning CD30 expression in PCGTCLs are not consistent [11,15], but, as was also expected, evidence of low-medium CD30 expression was found in 41% of our cases [10,16]. Although it may not correlate with prognosis, CD30 expression can be useful as a therapeutic target of the anti-CD30 monoclonal antibody brentuximab vedotin [16]. The combined use of brentuximab vedotin and gemcitabine as a bridge to alloSCT or after a relapse seems to increase patient survival, especially in chemo-resistant and aggressive cases [17,18]. This is encouraging because, although alloSCT remains the best means of providing long-term disease control, treatment outcomes vary: Isufi et al. [19] found that the 100-day transplant-related mortality was 12% among patients with PCGDTCL (*n* = 7) but as high as 29% among those with refractory MF/Sézary syndrome (SS) (*n* = 16).

The absence of CCR4 expression in PCGDTCLs hinders the use of the new anti-CCR4 monoclonal antibody mogamulizumab, which is now available for patients with MF/SS, but could also be useful in the differential diagnosis of PCGDTCL as MF is strongly CCR4 positive [5]. As in the case of the series of Jour et al. [20], the CCR4 negativity of our epidermotropic cases supported their classification as PCGDTCL. This also applied to the cases with MF-like lesions, although there was one notable exception of a patient with CCR4-positive MF-like plaques and a PCGDTCL non-epidermotropic nodulotumoral lesion (patient #19).

The expression of natural killer cell markers (CD16, CD56, and CD57) in PCGDTCL is possible and probably associated with the cell plasticity of the innate immune system [11]. However, CD56 expression varies and cannot be reliably used to diagnose PCGDTCL, although it has been previously reported in a case of subcutaneous PCGDTCL and, as alpha/beta subcutaneous lymphomas are usually CD56 negative, it may provide a useful clue for differential diagnosis [11,21,22]. In our cohort, CD56 positivity was found only in the patients with S-GDTCLs, and did not significantly correlate with survival.

In conclusion, the variable clinico-pathological scenarios of PCGDTCLs mean that it is still a challenging diagnosis. Moreover, recent evidence of the presence of gamma-delta T cells in the context of indolent cutaneous lymphomas suggests that the gamma-delta phenotype may not be aggressive by definition. However, our findings show that a subgroup of patients are characterised by an initially indolent MF-like phase that evolves into aggressive disease, and demonstrate the frequent occurrence of a change in the immunophenotype. Although the current WHO classification distinguishes gamma-delta MF from PCGDTCL, our findings support the idea that all of these entities should be classified together at least until additional prognostic markers become available.

## Figures and Tables

**Table 1:** Patients’ clinical characteristics.

Pts	Sex	Age	Subgroup	M/L	History of MF	Treatment	FU (mos)	Outcome
1	F	56	S-GDTCL	M	No	m-chemo	3	DOD
2	M	65	S-GDTCL	L	No	m-chemo	3	DOD
3	M	77	DE-GDTCL	M	Yes	m-chemo	5	DOD
4	M	46	S-GDTCL	L	No	m-chemo	12	LTF
5	M	50	S-GDTCL	M	No	m-chemo, brentuximab	14	DOD
6	M	52	S-GDTCL	M	No	m-chemo	6	DOD
7	F	72	DE-GDTCL	M	Yes	PUVA, RT, IFN	36	DOD
8	F	48	DE-GDTCL	M	No	PUVA, IFN, RT, bexa, m-chemo, alloSCT	228	DOO
9	M	47	DE-GDTCL	M	Yes	PUVA, bexa, s-chemo	84	AWD
10	M	76	S-GDTCL	M	No		12	DOD
11	F	76	DE-GDTCL	L	No		12	LTF
12	M	63	S-GDTCL	M	No	s-chemo	12	DOD
13	F	71	S-GDTCL	M	No	bexa, RT	10	DOD
14	M	70	S-GDTCL	M	No	s-chemo	60	AWD
15	F	64	S-GDTCL	M	No	m-chemo	24	DOD
16	M	65	DE-GDTCL	M	No	PUVA	3	DOO
17	F	59	S-GDTCL	M	No		3	LTF
18	F	60	DE-GDTCL	M	No	PUVA, bexa, s-chemo	168	DOD
19	F	64	DE-GDTCL	L	Yes	PUVA, RT	12	AWD
20	M	65	DE-GDTCL	M	No		12	DOD

S-GDTCL: subcutaneous GDTCL; DE-GDTCL: dermo-epidermal GDTCL; M/L: multiple or localised cutaneous lesions; m-chemo: multi-agent chemotherapy; s-chemo: single-agent chemotherapy; RT: local radiotherapy; IFN: interferon alpha; bexa: oral bexarotene; DOD: died of disease; DOO: died of other causes; AWD: alive with disease; LTF: lost to follow-up.

## Data Availability

The data presented in this study are available on request from the corresponding author.
